# Mid-titer human convalescent plasma administration results in suboptimal prophylaxis against SARS-CoV-2 infection in rhesus macaques

**DOI:** 10.3389/fimmu.2023.1085883

**Published:** 2023-02-10

**Authors:** Brandon J. Beddingfield, Nicholas J. Maness, Skye Spencer, Jay Rappaport, Pyone Pyone Aye, Kasi Russell-Lodrigue, Lara A. Doyle-Meyers, Robert V. Blair, HongMei Gao, David Montefiori, Chad J. Roy

**Affiliations:** ^1^ Divisions of Microbiology, Tulane National Primate Research Center, Covington, LA, United States; ^2^ Department of Microbiology and Immunology, Tulane School of Medicine, New Orleans, LA, United States; ^3^ Comparative Pathology, Tulane National Primate Research Center, Covington, LA, United States; ^4^ Veterinary Medicine, Tulane National Primate Research Center, Covington, LA, United States; ^5^ Duke Human Vaccine Institute, Duke University Medical Center, Durham, NC, United States; ^6^ Department of Surgery, Duke University Medical Center, Durham, NC, United States

**Keywords:** SARS-CoV-2, COVID-19, rhesus macaque, convalescent plasma, prophylaxis

## Abstract

**Introduction:**

SARS-CoV-2 is a respiratory pathogen currently causing a worldwide pandemic, with resulting pathology of differing severity in humans, from mild illness to severe disease and death. The rhesus macaque model of COVID-19 was utilized to evaluate the added benefit of prophylactic administration of human post-SARS-CoV-2 infection convalescent plasma (CP) on disease progression and severity.

**Methods:**

A pharmacokinetic (PK) study using CP in rhesus monkeys preceded the challenge study and revealed the optimal time of tissue distribution for maximal effect. Thereafter, CP was administered prophylactically three days prior to mucosal SARS-CoV-2 viral challenge.

**Results:**

Results show similar viral kinetics in mucosal sites over the course of infection independent of administration of CP or normal plasma, or historic controls with no plasma. No changes were noted upon necropsy via histopathology, although there were differences in levels of vRNA in tissues, with both normal and CP seemingly blunting viral loads.

**Discussion:**

Results indicate that prophylactic administration with mid-titer CP is not effective in reducing disease severity of SARS-CoV-2 infection in the rhesus COVID-19 disease model.

## Introduction

SARS-CoV-2, a pathogenic beta-coronavirus, is the cause of an ongoing worldwide pandemic. The disease resulting from infection by this virus, COVID-19, while largely presenting as a mild to moderate self-limiting respiratory illness, affects a percentage of individuals much more severely. This has resulted in almost six million deaths worldwide (1), and over one million deaths in the US (2), to date. The virus is highly transmissible as an airborne respiratory pathogen, with a low estimated infectious dose, making it highly successful at inducing large numbers of infections, often moving through populations rapidly. This has resulted in a large effort to produce an effective therapeutic or prophylaxis against infection or severe disease, in addition to the efforts toward production and distribution of vaccines.

Few options for prophylaxis and therapy were available early during the pandemic. One of the investigated options consisted of administration of convalescent plasma (CP) from individuals who recovered from prior infection by SARS-CoV-2. Some initial work indicated a potential for modulation of severe disease (3) and lowering viremia (4). This early promise led to clinical trials, specifically the Mayo Clinic’s COVID-19 Convalescent Expanded Access Program (EAP), eventually resulting in emergency use authorization from the FDA for administration to COVID-19 patients (5). Administration of CP has been correlated with lowered positivity by PCR for SARS-CoV-2 (6), and early delivery has been shown to reduce progression of disease (7). Accordingly, in designing this study, a prophylactic approach was selected initially to evaluate protective benefit of this source of CP before subsequent therapeutic assessments were performed.

Despite the early optimism surrounding CP administration, later analyses determined there to be no benefit, though there were no analyses performed on group subsets (8). The clinical trial focusing on emergency department CP treatment (NHLBI C3PO) was discontinued (9), and the RECOVERY trial showed no difference in 28-day mortality with or without treatment with CP (10). Many of these studies focused on administration of CP at a later time point, such as within 72 hours post symptom onset (8). This late time point administration may have a negative effect on the efficacy of CP therapy.

We hypothesize that administration of CP prior to SARS-CoV-2 challenge will maximize protective effect of the prophylactic intervention. Prior work has shown administration within 24 hours has a limited effect on viral shedding and clinical signs of disease (11). We utilize a nonhuman primate model of infection, shown before as susceptible to a mild to moderate disease process (12), to investigate the prophylactic administration of CP.

## Materials and methods

### Study approval

The Tulane University Institutional Animal Care and Use Committee approved all procedures used during this study. The Tulane National Primate Research Center (TNPRC) is accredited by the Association for the Assessment and Accreditation of Laboratory Animal Care (AAALAC no. 000594). The U.S. National Institutes of Health (NIH) Office of Laboratory Animal Welfare number for TNPRC is A3071-01. Tulane University Institutional Biosafety Committee approved all procedures for work in, and removal of samples from, Biosafety Level 3 laboratories.

### Virus and cells

Virus used for animal inoculation was strain SARS-CoV-2; 2019-nCoV/USA-WA1/2020 (BEI# NR-52281) prepared on subconfluent VeroE6 cells (ATCC# CRL-1586) and confirmed via sequencing. VeroE6 cells were used for live virus titration of biological samples and were maintained in DMEM (#11965092, Thermo Scientific, USA) with 10% FBS.

### Animals and procedures

A total of nine rhesus macaques of Indian origin (*Macaca mulatta*), between 3 and 11 years old, were utilized for this study. All rhesus macaques (RMs) were bred in captivity at TNPRC. For the PK study, three RMs were intravenously infused at standard rates with 4 mL/kg of human convalescent plasma (CP) obtained from prior, recovered SARS-CoV-2 infection or normal plasma (NP). Serum from RMs were monitored for RBD binding as well as neutralizing activity routinely for 68 days to determine pharmacokinetics. The NT_50_ of the CP used for both the PK and challenge studies was 1:1597 by pseudovirus neutralization assay. This plasma met the FDA recommended minimum neutralizing titer of CP to be used in therapy against SARS-CoV-2 of 1:160 (13). We define this as mid-titer plasma due to meeting the FDA recommended limit but falling below that of prior work selecting high titer plasma at levels of 1:3200 or above (7).

For the viral challenge study, four of the RMs were intravenously infused at standard rates with 4 mL/kg CP three days before challenge, with two RMs similarly infused with normal plasma. They were then exposed via intratracheal/intranasal (IT/IN) installation of viral inoculum (1mL intratracheal, 500 uL per nare, total delivery 2e+6 TCID_50_). Four historic controls of the same species and viral challenge dose, variant and route, are utilized for the purposes of comparisons in figures. Animal information, including plasma dosage and type, can be found in **Table 1**. Historic controls are listed as the final 4 animals.

**Table 1:** Study Animal Information.

Animal ID	Species	Age (years)	Source	Sex	Weight (kg)	Viral Dose (TCID_50_)	Plasma Dose (mL/kg)	Plasma Type
II67	*Macaca mulatta*	11	TNPRC	F	8.3	N/A	4	CP
JK23	*Macaca mulatta*	9	TNPRC	M	9.6	N/A	4	CP
L147	*Macaca mulatta*	5	TNPRC	M	9.1	N/A	4	CP
LI78	*Macaca mulatta*	5	TNPRC	M	8.2	2.0 X 10^6^	4	NP
LL28	*Macaca mulatta*	4	TNPRC	M	7.7	2.0 X 10^6^	4	NP
LC59	*Macaca mulatta*	6	TNPRC	M	7.5	2.0 X 10^6^	4	CP
IE32	*Macaca mulatta*	11	TNPRC	F	7.4	2.0 X 10^6^	4	CP
JJ76	*Macaca mulatta*	9	TNPRC	F	7.6	2.0 X 10^6^	4	CP
LJ15	*Macaca mulatta*	5	TNPRC	M	9.2	2.0 X 10^6^	4	CP
LM74	*Macaca mulatta*	4	TNPRC	M	6.2	2.0x106	N/A	N/A
IK92	*Macaca mulatta*	11	TNPRC	M	6.9	2.0 X 10^6^	N/A	N/A
KF89	*Macaca mulatta*	8	TNPRC	M	8.2	2.0 X 10^6^	N/A	N/A
LM30	*Macaca mulatta*	4	TNPRC	M	8.1	2.0 X 10^6^	N/A	N/A

N/A, not applicable.

The animals were monitored twice daily for the duration of the challenge study, with collections of mucosal swabs (nasal, pharyngeal, bronchial brush) as well as fluids (bronchoalveolar lavage) were taken pre-exposure as well as post-exposure days 1, 2, 3, 5 and at necropsy (or 1, 3 and necropsy for bronchial brush and BAL). For the PK study, BAL was performed through day 21 post infusion. Bronchial brushes were performed endoscopically. BAL consisted of instillation of 40mL of saline via feeding tube followed by removal via the same tube. Blood was collected pre-exposure, as well as 1, 2, 3, 5 and at necropsy for the challenge study, or up to day 68 post infusion for the PK study, in order to follow antibody levels. Physical examinations were performed daily after exposure, and necropsy occurred between 7- and 9-days post-exposure. During physical examination, rectal temperature and weight of each animal was performed. No animals met humane euthanasia endpoints during this study. Animals were euthanized at prescribed timepoints based upon experimental design of this evaluation. Animals were first anesthetized using ketamine and then administered euthanasia agent (Fatal plus, sodium pentobarbital, Lexington, KY). Death was confirmed by auscultation and absence of heartbeat. During necropsy, tissues were collected in media, fresh frozen, or in fixative for later analysis.

Prior to being assigned to the study, animals underwent the following: physical examination by a veterinarian, assessment of hematology and clinical chemistry, fecal direct and indirect examinations for intestinal parasites, and viral/pathogen screenings (including simian immunodeficiency virus (SIV), simian retrovirus type D (SRV), measles virus (MV), human papilloma virus 2 (HPV2), simian t-lymphotropic virus 1 (STLV1), SARS-CoV-2, *Mycobacterium tuberculosis, Burkholderia* sp.*, Shigella* sp., *Salmonella* sp., *Campylobacter sp*, *Escherichia coli*, *Tryapanosoma cruzi*, *Plasmodium* sp., and the study-specific pathogen SARS-CoV-2. Only animals considered healthy and determined to be free of screened pathogens were assigned to the study.

The animals underwent a one-week acclimation period following transfer to the ABSL3 facility prior to challenge for the purpose of allowing physiological and psychological stabilization before experimental manipulation. The TNPRC facilities are accredited by AAALAC International. Housing space requirements set forth by The Guide for the Care and Use of Laboratory Animals and the Animal Welfare Act are used to establish the minimum standard for housing all species at the TNPRC. Nonhuman primate standard caging dimensions are 4.3 ft^2^ x 36”H for those animals under 10kg, which included all animals under this study. The temperature set points for holding rooms for all nonhuman primates housed at the TNPRC ranged between 69-72°F, with a relative humidity target of 70%. Light cycle was set at 12:12 h of light:dark. All nonhuman primates were fed Purina LabDiet nonhuman primate diet, which is nutritionally complete. The Purina Mills diet was supplemented with a variety of fruits and vegetables at a minimum of three times each week. Water was provided *ad libitum*. For all procedures, animals were anesthetized per internal SOPs, with pain control occurring as per veterinary discretion.

### Quantification of Viral RNA in swab and tissue samples

Viral load in tissues, swabs and BAL cells and supernatant was quantified using RT-qPCR targeting the nucleocapsid (genomic and subgenomic) or envelope gene (subgenomic) of SARS- CoV-2. RNA was isolated from non-tissue samples using a Zymo Quick RNA Viral Kit (#R1035, Zymo, USA) or Zymo Quick RNA Viral Kit (#D7003, Zymo, USA) for BAL cells, per manufacturer’s instructions. RNA was eluted in RNAse free water. During isolation, the swab was placed into the spin column to elute the entire contents of the swab in each extraction. BAL supernatant was extracted using 100 µL, and serum was extracted using 500 µL. Viral RNA (vRNA) from tissues was extracted using a RNeasy Mini Kit (#74106, Qiagen, Germany) after homogenization in Trizol and phase separation with chloroform.

Isolated RNA was analyzed in a QuantStudio 6 (Thermo Scientific, USA) using TaqPath master mix (Thermo Scientific, USA) and appropriate primers/probes (14) with the following program: 25°C for 2 minutes, 50°C for 15 minutes, 95°C for 2 minutes followed by 40 cycles of 95°C for 3 seconds and 60°C for 30 seconds. Signals were compared to a standard curve generated using *in vitro* transcribed RNA of each sequence diluted from 10^8^ down to 10 copies. Positive controls consisted of SARS-CoV-2 infected VeroE6 cell lysate. Viral copies per swab were calculated by multiplying mean copies per well by amount in the total swab extract, while viral copies in tissue were calculated per microgram of RNA extracted from each tissue.

### Detection of neutralizing antibodies in serum

The ability of antibodies in serum to disrupt the binding of the receptor binding domain (RBD) of SARS-CoV-2 spike protein to Angiotensin Converting Enzyme (ACE_2_) was assessed via the Surrogate Virus Neutralization Test (GenScript# L00847) using the included kit protocol modified per the following: Serum samples were diluted from 1:10 to 1:21, 870 to determine an IC_50_ for RBD/ACE_2_ binding. Pseudovirus neutralization testing of matched serum was performed using a SARS-CoV-2 D614G spike-pseudotyped virus in 293/ACE_2_ cells, with neutralization assessed via reduction in luciferase activity (15, 16). Pseudovirus assay was utilized for both determination of RM plasma titers over time as well as CP material prior to RM infusion.

### Statistical analysis

Comparisons between the area under the curve measurements of vRNA were made using ANOVA with Geisser-Greenhouse correction and Holm-Šídák multiple comparisons test. Time-based comparisons of vRNA were performed using a two-way ANOVA with Geisser-Greenhouse correction and a Tukey multiple comparisons test.

## Results

### Antibody levels wane rapidly following CP administration

**Figure 1:**
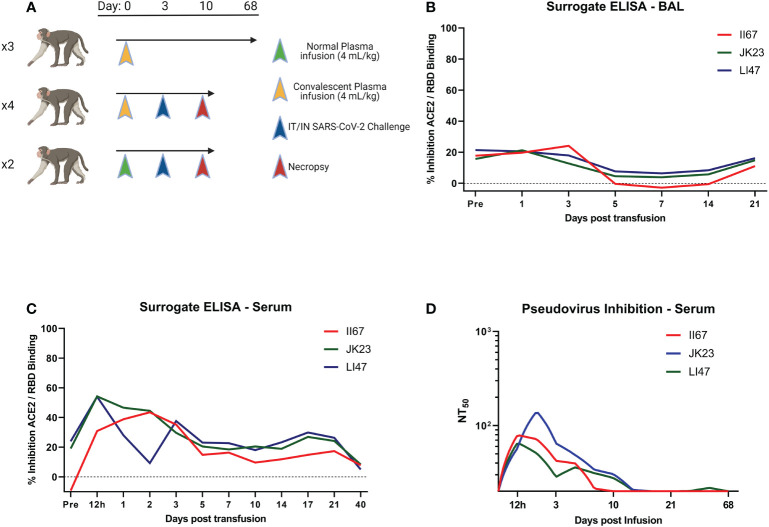
Study Design and Antibody Kinetics. **(A)** Three RMs were given 4 mL/kg CP and followed for 68 days to determine pharmacokinetics. Following this, 6 RMs were given either NP or CP and challenged with SARS-CoV-2 three days later. **(B, C)** Surrogate ELISA was used to follow plasma kinetics of BAL and serum, respectively. **(D)** Serum kinetics were followed by pseudovirus inhibition assay. Study design made in Biorender.

We infused three RMs with convalescent plasma and followed antibody levels long term for pharmacokinetic determination (**Figure 1A**). Using a surrogate ELISA examining the ability of antibody to disrupt RBD/ACE_2_ interaction, we determined antibody levels were higher overall in BAL on day 3 post infusion, but wane in both BAL and serum rapidly (**Figures 1B, C**). Pseudovirus inhibition assay performed on serum shows a similar pattern, with levels falling to baseline in one individual by day 10 post infusion (**Figure 1D**). Based on this data, a separate set of RMs were challenged with SARS-CoV-2 three days post infusion with normal plasma (NP) or convalescent plasma (**Figure 1A**). One day post challenge, ID_50_ levels were between 1:40 and 1:82 (data not shown).

### Mucosal viral RNA content shows mild reductions with use of CP

**Figure 2:**
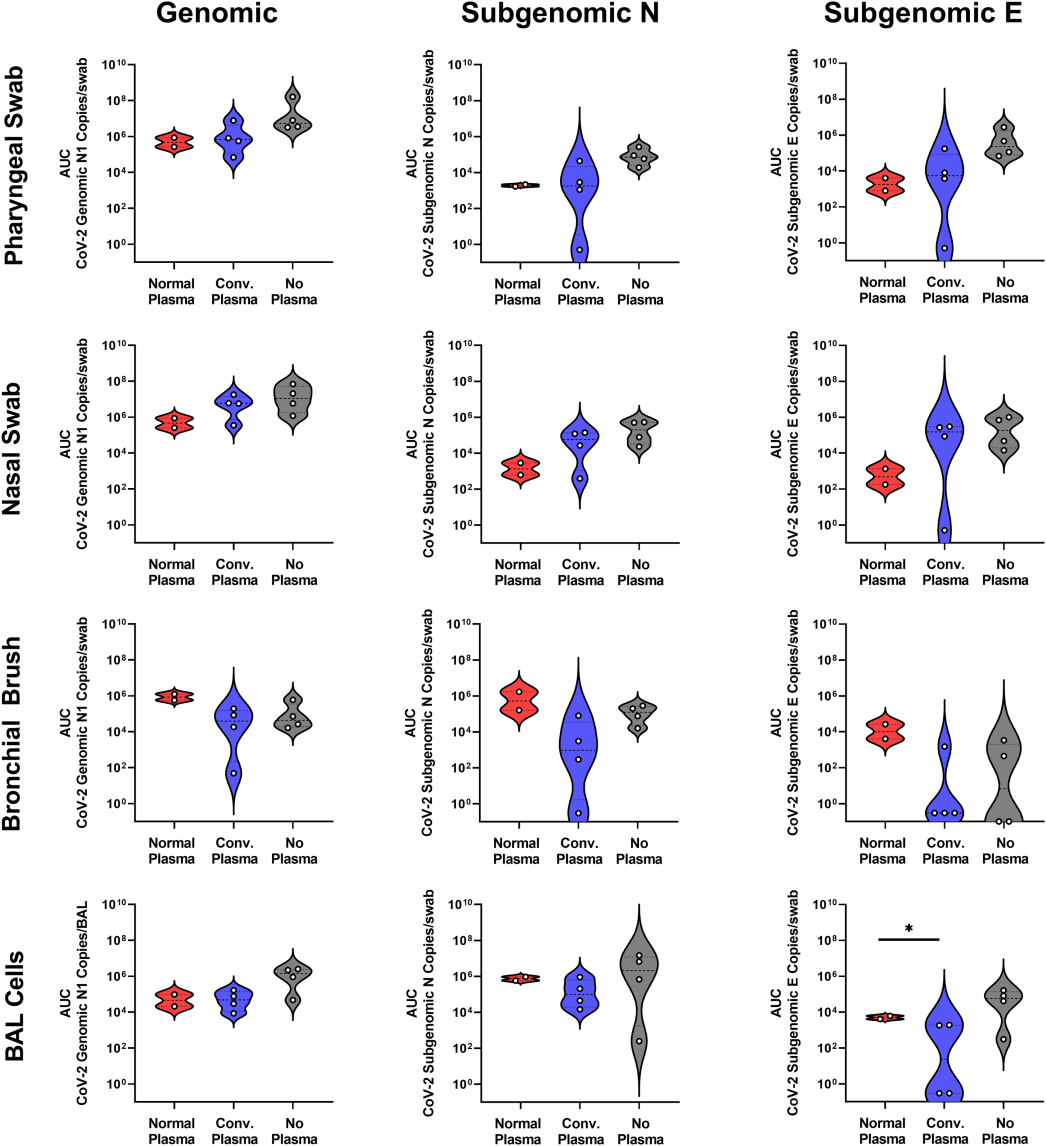
Mucosal vRNA Content post SARS-CoV-2 Challenge. Viral loads, measured via genomic N and subgenomic (E and N) vRNA content, were determined in pharyngeal and nasal swabs, as well as bronchial brushes and BAL cells. Data is represented as area under the curve of vRNA content over the course of the study. Groups were compared via ANOVA with Geisser-Greenhouse correction and Holm-Šídák multiple comparisons test (*p<0.05).

We challenged a cohort of RMs with 2x10^6^ TCID_50_ SARS-CoV-2 and followed viral loads via qPCR using nasal and pharyngeal swabs, bronchial brushes, and BAL cell isolation, as these are the primary sites of infection for this virus. No differences were found between the AUC of viral genomic or subgenomic content of RMs administered normal or convalescent plasma except for BAL cell subgenomic E content (**Figure 2**). No differences were found between those two groups and historic controls administered no plasma at all (**Figure 2**). Some sites, including bronchial brush, show a trend toward a reduction in vRNA in individuals administered CP, indicating a potential effect that is not significant due to small sample size.

**Figure 3:**
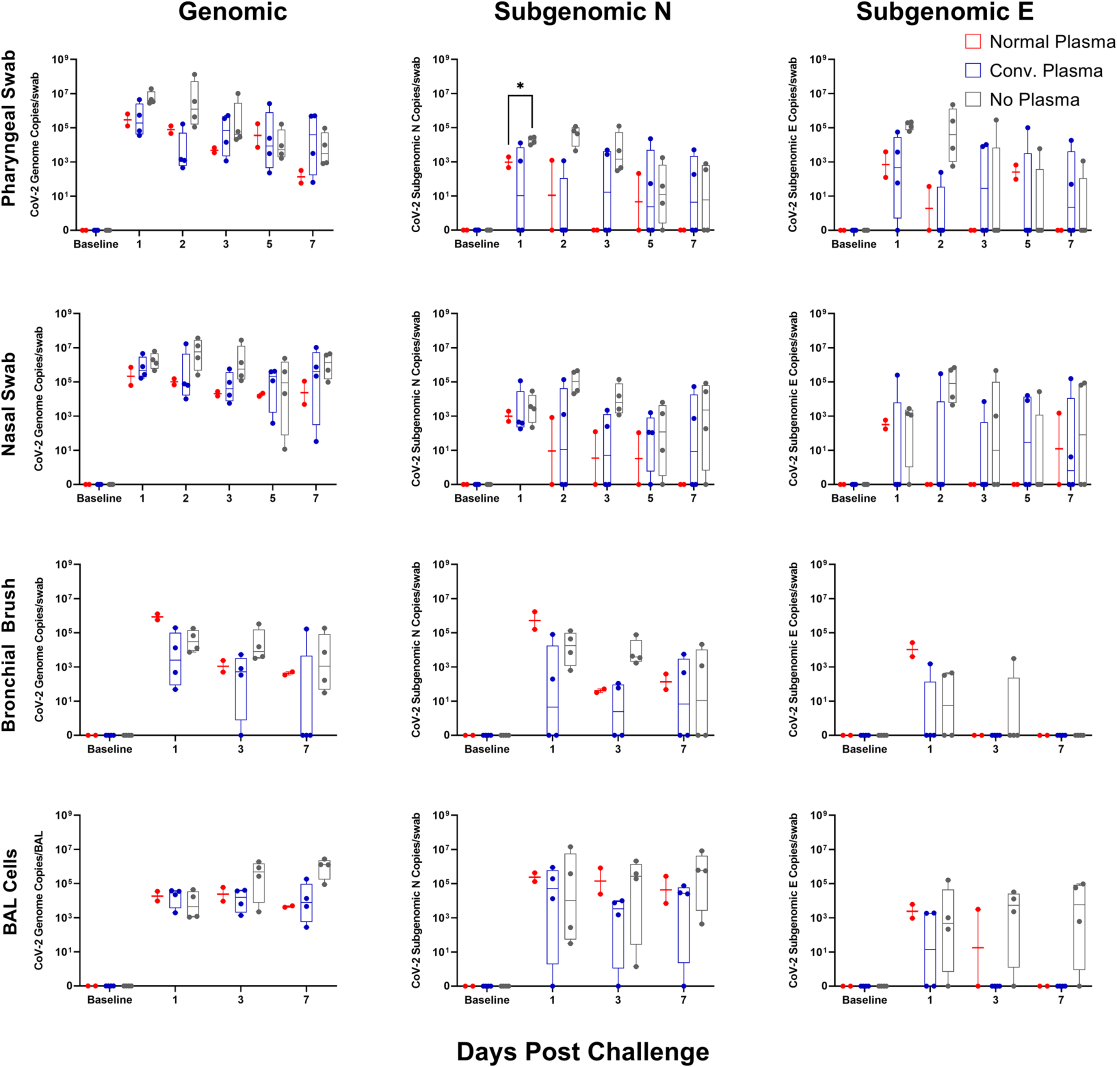
Mucosal vRNA Content post SARS-CoV-2 Challenge. Viral loads, measured via genomic N and subgenomic (E and N) vRNA content, were determined in pharyngeal and nasal swabs, as well as bronchial brushes and BAL cells. Data is represented as copies per swab or BAL of vRNA content per collection timepoint. Groups were compared via two-way ANOVA with Geisser-Greenhouse correction and a Tukey multiple comparisons test (*p<0.05).

Viral RNA day-by-day shows an increase in subgenomic N content one day post challenge in the cohort administered no plasma as compared to that administered normal plasma. No other significant differences were seen, though there were patterns indicative of slight effect (**Figure 3**). Viral RNA content was blunted earlier in the bronchi upon CP administration compared to normal plasma, but did not reach significance, likely again due to a small sample size (**Figure 3**). Genomic and subgenomic content persisted longer in the pharyngeal swabs in both the CP and no plasma cohorts (**Figure 3**).

### Tissue viral RNA content at necropsy is similar between normal plasma and CP

**Figure 4:**
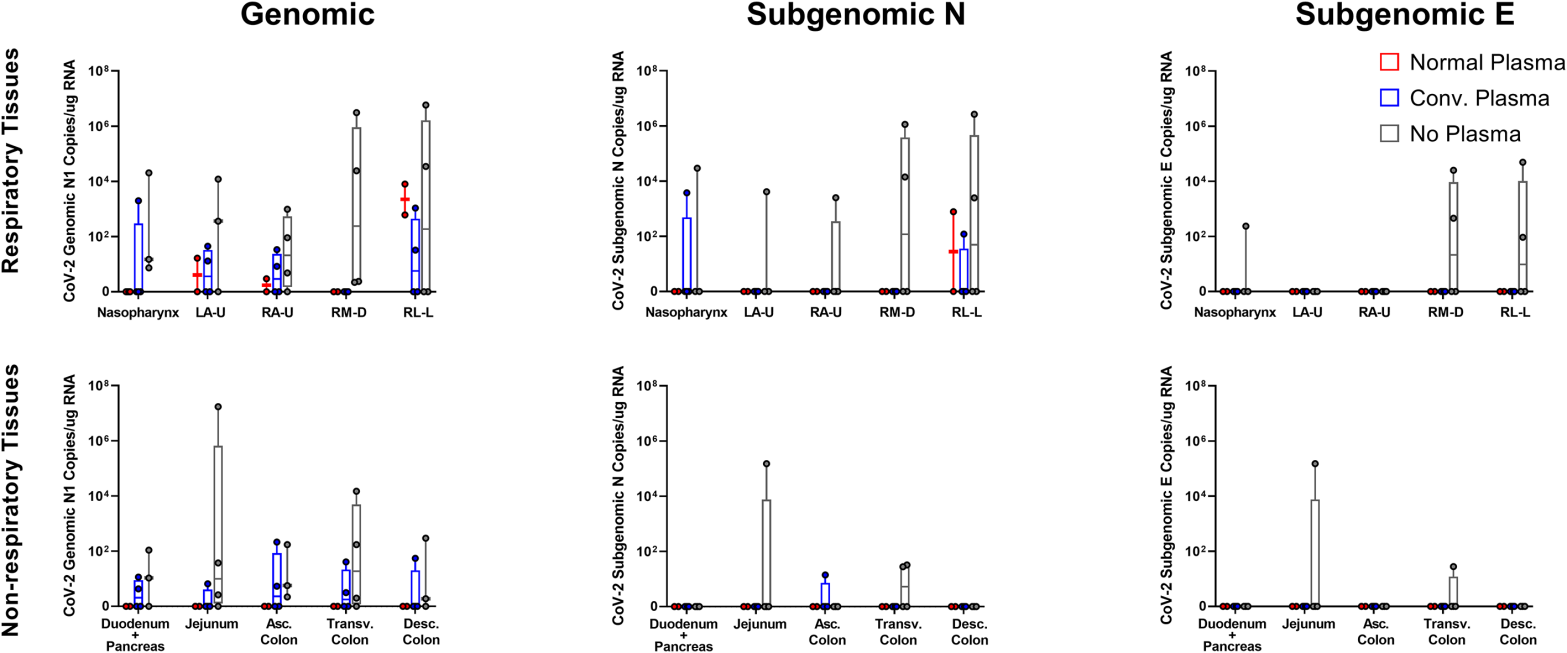
Tissue vRNA Content post SARS-CoV-2 Challenge. Viral loads, measured via genomic N and subgenomic (E and N) vRNA content, were determined in respiratory and digestive tissues, as well as bronchial brushes and BAL cells. Data is represented as copies per ug extracted RNA at necropsy.

After necropsy, vRNA content was examined in respiratory and gastrointestinal (GI) tissues. In respiratory sites, genomic and subgenomic content was generally lower in both NP and CP animals than the no plasma cohort. Gastrointestinal sites showed higher genomic vRNA loads in the CP cohort than the NP cohort, but less than the no plasma cohort. Very little subgenomic N vRNA was found in the GI tract, with the same pattern being displayed. No subgenomic E content was found in either NP or CP cohorts for either respiratory or gastrointestinal sites (**Figure 4**).

In addition to vRNA content of tissues, administration of CP did not alter the clinical course of SARS-CoV-2 infection. No significant disease was observed in either treated or untreated animals beyond mild respiratory signs that were not significantly different between cohorts.

## Discussion

Convalescent plasma therapy has been used for infectious diseases for over a century since von Behring developed the practice in the late 19^th^ century. Early on mostly utilized for bacterial diseases, it has also been used against viral infections spread by the respiratory route including influenza and measles (17–20). Mechanisms thought to be protective include neutralization that mitigates viral burden as well as non-neutralizing Fc-based antibody functions that reduce lung inflammation (21). Due to the novel nature of SARS-CoV-2 at the beginning of the worldwide pandemic, CP was explored as a therapeutic approach in the absence of any available virus-specific antiviral or vaccine. Accordingly, we tested the prophylactic potential of CP administered three days prior to infection with SARS-CoV-2. We demonstrate that CP that is of insufficient titer does not alter most viral kinetics in the host. This data underscores the lack of utility of CP in the prophylaxis of COVID-19.

Antibodies capable of RBD/ACE2 binding inhibition or neutralization were highest in BAL at three days post administration and between 12 hours and three days post administration in serum, though particularly high levels were never achieved, even with the more sensitive pseudovirus inhibition assay. Human antibody kinetics in macaques are skewed toward shorter half-lives, though this 3-day time frame is not of sufficient length for that be a driving factor of efficacy in this study (22). Viral kinetics in mucosal sites were similar regardless of administration of NP or CP. Tissue viral loads showed differences, with NP and CP both seemingly able to lower tissue vRNA content one week post challenge, especially in the subgenomic content. There was nothing significant noted in histopathology at necropsy (data not shown).

This work agrees with earlier work in the nonhuman primate model that focused on CP administration soon after SARS-CoV-2 challenge, also utilizing mid-titer CP (11). Lack of histopathological modification, viral kinetics, or changes in development of later immune responses such as antibody development indicates lack of disease modification capacity whether given early after infection, or even before infection. Treatment early post infection with high titer CP in another nonhuman primate model did produce a potentially clinical benefit, with reduced lung pathology and viral loads seen (23). This indicates a high degree of variability in therapeutic efficacy between pools of CP, leading to less certainty as to the effective nature of any given CP lot.

In contrast, prophylaxis by potent, long half-life, neutralizing monoclonal antibodies has been shown in this model of SARS-CoV-2 infection to reduce viral loads at mucosal sites up to 75 days post administration (14). With multiple lots of a monoclonal antibody (mAb) preparation being much more standardized than a CP preparation (24), this makes therapeutics consisting of mAbs much more appealing. Indeed, trials regarding therapy via CP have been discontinued due to lack of apparent efficacy (9, 10). With the advent of robust vaccines and antiviral medications, CP therapy and prophylaxis are no longer an avenue of significant exploration regarding infection by SARS-CoV-2.

## Data availability statement

The raw data supporting the conclusions of this article will be made available by the authors, without undue reservation.

## Ethics statement

The animal study was reviewed and approved by Tulane IACUC.

## Author contributions

All authors listed have made a substantial, direct, and intellectual contribution to the work and approved it for publication.

## References

[1] Center for systems science and engineering. In: . Johns Hopkins University. Available at: https://coronavirus.jhu.edu/map.html. CoVID-19 Dashboard Johns Hopkins University: Johns Hopkins University.

[2] Centers for disease control and prevention. In: COVID data tracker 2021. Available at: https://covid.cdc.gov/covid-data-tracker/#datatracker-home.

[3] Shen C Wang Z Zhao F Yang Y Li J Yuan J. Treatment of 5 critically ill patients with COVID-19 with convalescent plasma. JAMA. (2020) 323(16):1582–9. doi: 10.1001/jama.2020.4783 PMC7101507 32219428

[4] Duan K Liu B Li C Zhang H Yu T Qu J. Effectiveness of convalescent plasma therapy in severe COVID-19 patients. Proc Natl Acad Sci U S A (2020) 117(17):9490–6. doi: 10.1073/pnas.2004168117 PMC7196837 32253318

[5] Joyner MJ Carter RE Senefeld JW Klassen SA Mills JR Johnson PW. Convalescent plasma antibody levels and the risk of death from covid-19. N Engl J Med (2021) 384(11):1015–27. doi: 10.1056/NEJMoa2031893 PMC7821984 33523609

[6] Li L Zhang W Hu Y Tong X Zheng S Yang J. Effect of convalescent plasma therapy on time to clinical improvement in patients with severe and life-threatening COVID-19: A randomized clinical trial. JAMA. (2020) 324(5):460–70. doi: 10.1001/jama.2020.10044 PMC7270883 32492084

[7] Libster R Perez Marc G Wappner D Coviello S Bianchi A Braem V. Early high-titer plasma therapy to prevent severe covid-19 in older adults. N Engl J Med (2021) 384(7):610–8. doi: 10.1056/NEJMoa2033700 PMC7793608 33406353

[8] Janiaud P Axfors C Schmitt AM Gloy V Ebrahimi F Hepprich M. Association of convalescent plasma treatment with clinical outcomes in patients with COVID-19: A systematic review and meta-analysis. JAMA. (2021) 325(12):1185–95. doi: 10.1001/jama.2021.2747 PMC7911095 33635310

[9] Korley FK Durkalski-Mauldin V Yeatts SD Schulman K Davenport RD Dumont LJ. Early convalescent plasma for high-risk outpatients with covid-19. N Engl J Med (2021) 385(21):1951–60. doi: 10.1056/NEJMoa2103784 PMC8385553 34407339

[10] Group RC. Convalescent plasma in patients admitted to hospital with COVID-19 (RECOVERY): a randomised controlled, open-label, platform trial. Lancet. (2021) 397(10289):2049–59. doi: 10.1016/S0140-6736(21)00897-7 PMC8121538 34000257

[11] Deere JD Carroll TD Dutra J Fritts L Sammak RL Yee JL. SARS-CoV-2 infection of rhesus macaques treated early with human COVID-19 convalescent plasma. Microbiol Spectr (2021) 9(3):e0139721. doi: 10.1128/Spectrum.01397-21 34817208 PMC8612156

[12] Mercado NB Zahn R Wegmann F Loos C Chandrashekar A Yu J. Single-shot Ad26 vaccine protects against SARS-CoV-2 in rhesus macaques. Nature. (2020) 586(7830):583–8. doi: 10.1038/s41586-020-2607-z PMC7581548 32731257

[13] Root HB Gilleskie M Lu CH Gilmore A Evans M Nelson BG. Evaluation of a COVID-19 convalescent plasma program at a U.S. academic medical center. PloS One (2022) 17(12):e0277707. doi: 10.1371/journal.pone.0277707 36480499 PMC9731422

[14] Beddingfield BJ Maness NJ Fears AC Rappaport J Aye PP Russell-Lodrigue K. Effective prophylaxis of COVID-19 in rhesus macaques using a combination of two parenterally-administered SARS-CoV-2 neutralizing antibodies. Front Cell Infect Microbiol (2021) 11:753444. doi: 10.3389/fcimb.2021.753444 34869063 PMC8637877

[15] Shen X Tang H McDanal C Wagh K Fischer W Theiler J. SARS-CoV-2 variant B.1.1.7 is susceptible to neutralizing antibodies elicited by ancestral spike vaccines. Cell Host Microbe (2021) 29(4):529–39 e3. doi: 10.1016/j.chom.2021.03.002 33705729 PMC7934674

[16] Weissman D Alameh MG de Silva T Collini P Hornsby H Brown R. D614G spike mutation increases SARS CoV-2 susceptibility to neutralization. Cell Host Microbe (2021) 29(1):23–31 e4. doi: 10.1016/j.chom.2020.11.012 33306985 PMC7707640

[17] Casadevall A Dadachova E Pirofski LA. Passive antibody therapy for infectious diseases. Nat Rev Microbiol (2004) 2(9):695–703. doi: 10.1038/nrmicro974 15372080

[18] Luke TC Kilbane EM Jackson JL Hoffman SL. Meta-analysis: convalescent blood products for Spanish influenza pneumonia: a future H5N1 treatment? Ann Intern Med (2006) 145(8):599–609. doi: 10.7326/0003-4819-145-8-200610170-00139 16940336

[19] Gallagher JR. Use of convalescent measles serum to control measles in a preparatory school. Am J Public Health Nations Health (1935) 25(5):595–8. doi: 10.2105/AJPH.25.5.595 PMC1559172 18014217

[20] Franchini M. Convalescent plasma therapy for managing infectious diseases: a narrative review. Ann Blood (2021) 6:17. doi: 10.21037/aob-2020-cp-03

[21] Winkler ES Gilchuk P Yu J Bailey AL Chen RE Chong Z. Human neutralizing antibodies against SARS-CoV-2 require intact fc effector functions for optimal therapeutic protection. Cell. (2021) 184(7):1804–20 e16. doi: 10.1016/j.cell.2021.02.026 33691139 PMC7879018

[22] Hinton PR Johlfs MG Xiong JM Hanestad K Ong KC Bullock C. Engineered human IgG antibodies with longer serum half-lives in primates. J Biol Chem (2004) 279(8):6213–6. doi: 10.1074/jbc.C300470200 14699147

[23] Cross RW Prasad AN Borisevich V Woolsey C Agans KN Deer DJ. Use of convalescent serum reduces severity of COVID-19 in nonhuman primates. Cell Rep (2021) 34(10):108837. doi: 10.1016/j.celrep.2021.108837 33662255 PMC7901292

[24] Bordeaux J Welsh A Agarwal S Killiam E Baquero M Hanna J. Antibody validation. Biotechniques. (2010) 48(3):197–209. doi: 10.2144/000113382 20359301 PMC3891910

